# Emotional Mirror Neurons in the Rat’s Anterior Cingulate Cortex

**DOI:** 10.1016/j.cub.2019.05.064

**Published:** 2019-06-17

**Authors:** Maria Carrillo, Yinging Han, Filippo Migliorati, Ming Liu, Valeria Gazzola, Christian Keysers

(Current Biology *29*, 1301–1312.e1–e6; April 22, 2019)

It has come to our attention that this article’s STAR Methods section was missing information regarding the anesthetics and analgesics used for the surgical procedures, as well as animal welfare-related information, as recommended by the ARRIVE guidelines. This missing information, which does not affect our results or conclusions, has now been added in the article online. The authors apologize for the oversight.

